# Imaging Quality Evaluation of Low Tube Voltage Coronary CT Angiography Using Low Concentration Contrast Medium

**DOI:** 10.1371/journal.pone.0120539

**Published:** 2015-03-26

**Authors:** Chengzhong Zhang, Yuejun Yu, Zaixian Zhang, Qingguo Wang, Linfeng Zheng, Yan Feng, Zhiguo Zhou, Guixiang Zhang, Kangan Li

**Affiliations:** 1 Department of Radiology, Shanghai General Hospital, Shanghai Jiaotong University School of Medicine, Shanghai, People’s Republic of China; 2 Department of Interventional Radiology, Weifang People's Hospital, Weifang, Shandong, People’s Republic of China; Northwestern University Feinberg School of Medicine, UNITED STATES

## Abstract

**Purpose:**

To compare the image quality of prospectively ECG-gated low voltage coronary computed tomography angiography (CTA) with an administration of low concentration contrast medium.

**Method and Materials:**

A total of 101 patients, each with a heart rate below 65 beats per minute (BPM), underwent a prospectively ECG-gated axial scan in CT coronary angiography on a 64-slice CT scanner. All patients were allocated in three groups (group A: n=31, 80kVp, 300 mgI/ml; group B: n=34, 100kVp, 300 mgI/ml; group C: n=36, 120kVp, 370 mgI/ml). The CT attenuation values of aortic root (AR), left main coronary artery (LMA), right main coronary artery (RMA) and chest subcutaneous fat tissue were measured. The contrast-to-noise ratio (CNR) of AR, LMA and RMA were calculated according to the formulas below. The values of computed tomography dose index (CTDI) and dose-length product (DLP) were recorded. Image quality was assessed on a 5-point scale. The results were compared using the one-way ANOVA and rank sum tests.

**Results:**

The values of CNR and SNR for vessels in group A and group B were not significantly different from group C (each p > 0.05). The effective radiation dose in group A (1.51±0.70 mSv) and group B (2.59±1.24 mSv) were both lower than group C (4.92±2.82 mSv) (each p < 0.05). There was no significant difference among the image quality scores of group A (4.10±0.41), group B (3.90±0.48) and group C (4.04±0.36) (each P > 0.05).

**Conclusion:**

Low tube voltage coronary CT angiography using low concentration contrast medium does not affect the imaging quality for assessing the coronary arteries compared with high voltage coronary CT angiography using high concentration contrast medium. Meanwhile low concentration contrast medium allowed 47-69% of radiation dose reduction.

## Introduction

Multi-detector-row computed tomography (MDCT) has been considered as the method of choice for the diagnosis, treatment planning and follow-up of many diseases, such as coronary heart disease (CHD), myocardial infarction (MI) and pulmonary embolism (PE). CT angiography (CTA) uses MDCT for rapid continuous scanning and, through multiple-planar and three-dimensional CT restructuring, is able to obtain a set of vascular images. Technical advances, such as the addition of detector rows, have significantly improved the spatial resolution and scanning speed of MDCT, which makes MDCT improve the diagnostic accuracy for coronary artery stenosis [1–3]. Despite those advantages compared to conventional diagnostic workup, radiation exposure is still a major concern [4]. It is reported that it produces 11–15 mSv to 21 mSv at 64-silice MDCT to CT coronary angiography [5–7] On the other hand, the concentration of the contrast agent plays an important role in image quality, which may affect the diagnosis of vascular disease, including CHD. A high concentration of contrast medium can help generate high quality images of the coronary arteries but may also hide calcified plaque which is an important factor to make a diagnosis of CHD [8] and cause the contrast nephropathy which can lead to acute renal failure [9–12], particularly in patients with preexisting renal impairment associated with diabetes [13]. Therefore, maintaining image quality with a minimal amount of contrast agent and radiation dose is of significant importance for clinical practice. Many reports have compared contrast media with different iodine concentrations during the enhancement of multiple organs and vessels on CT scans [14–17].

Additionally, because dose and radiation exposure vary approximately with the square of the voltage under the constant tube current, lowering the voltage has a greater influence on patient dose than dose reducing the tube current [18]. Weilan Zhang found that with the help of iterative reconstruction algorithm techniques, head-and-neck CTA of diagnostic quality can be adequately acquired with low tube voltage and low concentration contrast media [19]. However, a few studies demonstrates that the relatively low voltage has an impact on computed tomography CT coronary angiography images when acquired using a low concentration of contrast agent [20,21]. Thus, the purpose of our study is to evaluate two types of different concentrations of iodine contrast agent at different voltages in order to determine whether a lower concentration of contrast agent with relatively low kVp can result in the same image quality.

## Materials and Methods

### Study design

This study was approved by Shanghai General Hospital Institutional Review Board. All patients were informed of the possible adverse effects of the two types of contrast media—in advance and by clinical doctors—and signed an informed consent form. We included 101 consecutive patients scheduled for CT coronary angiography and recorded their sex, age, height (m), weight (kg) and body mass index (BMI, weight/height^2^) from February 11, 2014 to April 25, 2014. Inclusion criteria included patients referred for CT coronary angiography due to chest pain or suspected coronary artery diseases. Exclusion criteria included patients who had arrhythmia, renal insufficiency, allergic reaction to iodine contrast medium, previous history of surgery or stenting for coronary artery diseases, heart failure, and women who were potentially pregnant or nursing. Additionaly, patients who were unable to cooperate with breath-holding for at least 10 s or had a BMI≥28 were not enrolled in the study (few patients who had a BMI≥28 and they were considered overweight), either. As a prospectively ECG-gated axial scan in CT coronary angiography on a 64-slice CT scanner requires a relatively slow heart rate, only patients with heart rate below 65 BMP were included (patients were not given Beta-blockers). They received contrast medium with either 300 mg I/ml or 370 mg I/ml during the examination at random.

All the patients were allocated in three groups: 31 patients in group A (17 males and 14 females) had their CT coronary angiography scanned at 80kVp using low concentration contrast medium (300 mg iodine (I)/ml); 34 patients in group B (18 males and 16 females, 23**≤**BMI<28) had their CT coronary angiography scanned at 100kVp using low concentration contrast medium (300 mg iodine (I)/ml) and 36 patients in group C (26 males and 10 females) had their CT coronary angiography scanned at 120kVp using high concentration contrast medium (370 mg iodine (I)/ml).

### Contrast medium infusion protocols and CT scanning

The contrast medium was kept at 37.5°C and injected with an 18-gauge needle through the right elbow vein by the use of a binocular high-pressure syringe (Irich, medical, German). The amount of contrast was 1 ml/kg, administered at a flow rate of 4 ml/s [22]. All patients were examined on a 64-row detector system (Discovery CT750 HD, GE healthcare) in the craniocaudal direction to cover from the AR to the caudal end of the heart with the following settings: beam collimation, 64×0.625 mm; rotation time, 0.35 s; section thickness and intervals, 0.625 mm; image matrix, 512×512; and field of view, 32 cm. Automatic tube current modulation for 80 kVp was in the range of 100 mA to 700 mA; the tube current at 100 kVp was in the range of 150 mA to 660 mA; the tube current at 120 kVp was in the range of 100 mA to 550 mA. Then, patients were examined by cardiac scan and enhanced scanning. Contrast medium was injected followed by a flush of46 ml-55 ml 0.9% chloride sodium [Baxter, Baxter Healthcare (Shanghai) CO.LTD]. Data from Group A and group B were both subjected to adaptive statistical iterative reconstruction (ASIR). Data from the traditional group C adopted filter back projection (FBP) reconstruction.

### Radiation dose

The dose-length product (DLP) and computed tomography dose index (CTDI) displayed on the CT system were recorded to calculate the radiation dose by the formulas: radiation dose = DLP*0.014 [23].

### Image post-processing and data collection

The acquisition phase window was a quarter of the R-R period. Reconstruction of the main method packages includes multi-planar reconstruction (MPR), maximum density projection (MIP) and volume rendering (VR).

The images were analyzed by an experienced cardiac radiologist who was blinded to the contrast medium used. Axial sections were selected to measure the mean CT attenuation values of AR, LMA and RMA by placing an ROI in the center of these parts. The mean CT attenuation values of chest subcutaneous fat tissue were also recorded as background contrast. All ROIs were made as large as possible according to vessel sizes, but vessel walls, calcifications or metallic artifacts were avoided to prevent partial volume effects. Image noise was defined as the mean standard deviation of the chest subcutaneous fat tissue. The contrast-to-noise ratio (CNR) and signal noise ratio (SNR) of AR, LMA and RMA were calculated according to the formulas: CNR = (C_V_-C_B_)/N; SNR = C_V_/N; C_V_ represents mean CT attenuation values of AR, LMA or RMA, C_B_ represents mean CT attenuation values of chest subcutaneous fat tissue, N represents image noise. CNR and SNR were regarded as an objective evaluation of the image quality.

Image quality was rated axially by two radiologists, each with more than 5 years of experience in assessing coronary angiography imaging; each radiologists was blinded to all scanning and processing conditions. Image quality of LMA, proximal, middle and distal parts of left anterior descending (LAD), proximal and distal left circumflex (LC) and proximal, middle and distal parts of RMA was assessed on 5-point scale: 5 = excellent with no granularity, no or minimal artifacts and clear vessel display; 4 = between 5 and 3; 3 = acceptable for diagnosis, but small amounts of radiation and high density or irregular low density artifacts and fuzzy boundaries were present; 2 = suboptimal with some artifacts, but acceptable; 1 = diagnostic decision cannot be made due to blurry boundaries and large amounts of radiation, high density or irregular low density artifacts. In case of disagreement, final decisions were reached by consensus.

### Statistical Analysis

All statistical analyses were performed with a statistical software package (SAS version9.0; SAS Software, Cary, USA). Data were shown as the mean ± standard deviation. All measurement data results were compared with one-way ANOVA (such as age, radiation dose and mean CT attenuation values), all ranked data were compared with rank sum test (such as image scores). For all statistical analyses, p<0.05 was considered significant.

## Results

### Demographic characteristics

Of the 101 patients initially recruited for this study, 31 patients were assigned to group A, 34 patients were assigned to group B and 36 patients were assigned to group C. There were 61 men and 40 women (age range = 41–81 years; median age = 60 years). The demographics and characteristics of the patients are displayed in Table 1, and pairwise comparisons are displayed in Table 2. We found no significant differences among the three groups in terms of number, gender, age and height. There were significant differences among the three groups in terms of weight and BMI.

**Table 1:** Characteristics of three groups.

character	overall	A	B	C	P value
Total no.	101	31	34	36	-
Male/Female	101	17/14	18/16	26/10	0.19
Age (years)	-	60.51±7.22	61.32±7.17	59.39±8.53	0.57
Height (m)	-	1.68±0.09	1.66±0.07	1.69±0.08	0.38
Weight (kg)	-	62.00±6.95	70.09±8.17	71.44±7.74	<0.0001
BMI (kg/m^2^)	-	21.89±1.37	25.24±1.45	24.99±1.84	<0.0001
CTDI (mGy)	-	7.85±2.71	14.04±5.40	25.07±10.62	<0.05
DLP (mGy cm)	-	107.90±50.04	185.10±88.75	351.97±201.75	<0.05
Radiation dose (mSv)	-	1.51±0.70	2.59±1.24	4.92±2.82	<0.05
C_A_(HU)	-	565.31±100.03	439.27±87.47	336.83±50.51	<0.05
C_L_(HU)	-	606.46±102.48	451.26±79.20	356.88±57.72	<0.05
C_R_(HU)	-	597.31±111.80	439.31±90.42	353.15±62.85	<0.05
N	-	22.88±7.30	19.06±6.14	15.61±4.89	<0.05
CNR_A_	-	34.63±15.34	32.12±10.80	32.53±11.65	0.70
SNR_A_	-	28.18±12.94	25.30±8.91	23.96±8.93	0.24
CNR_L_	-	36.67±16.31	33.02±11.71	33.76±11.77	0.51
SNR_L_	-	30.22±13.94	26.20±9.80	25.18±9.06	0.15
CNR_R_	-	36.19±16.13	32.14±11.30	33.60±12.01	0.46
SNR_R_	-	29.74±13.85	25.32±9.33	25.02±9.29	0.16

**Table 2:** Pairwise comparisons of patients’ characteristics.

character	A vs B	A vs C	B vs C
Weight (kg)	<0.05	<0.05	0.45
BMI (kg/m^2^)	<0.05	<0.05	0.51
CTDI (mGy)	<0.05	<0.05	<0.05
DLP (mGy cm)	<0.05	<0.05	<0.05
Radiation dose (mSv)	<0.05	<0.05	<0.05
C_A_(HU)	<0.05	<0.05	<0.05
C_L_(HU)	<0.05	<0.05	<0.05
C_R_(HU)	<0.05	<0.05	<0.05
N	<0.05	<0.05	<0.05

### Radiation dose analysis

**Fig 1:**
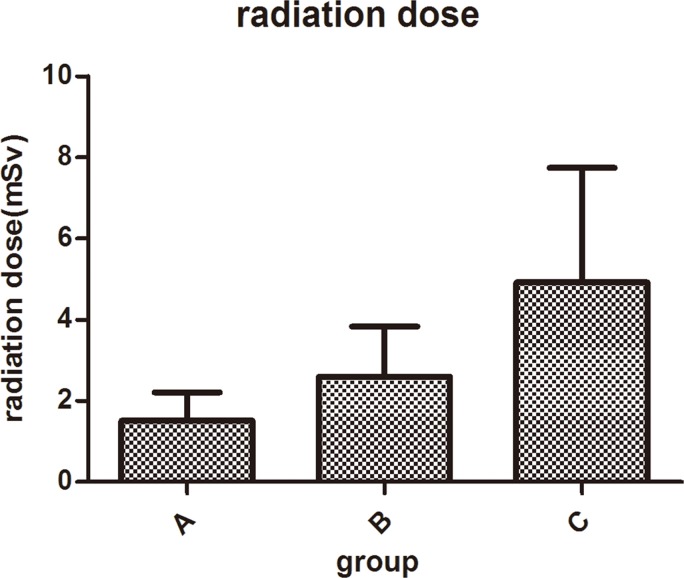
Patients’ radiation dose data. Patients’ radiation dose data of three groups(P<0.0001). It shows that radiation dose of group A is the least and group C is the most.

The radiation dose data for the patients are displayed on Table 1, and pairwise comparisons are displayed in Table 2. Significant differences were found among three groups in terms of CTDI, DLP and radiation dose. We found that radiation doses of group A (1.51±0.70 mSv) and group B (2.60±1.28 mSv) are much lower than that of group C (4.96±2.86 mSv), as shown in Fig. 1. The radiation dose reduction was 47–69%.

### Objective image quality analysis

**Fig 2:**
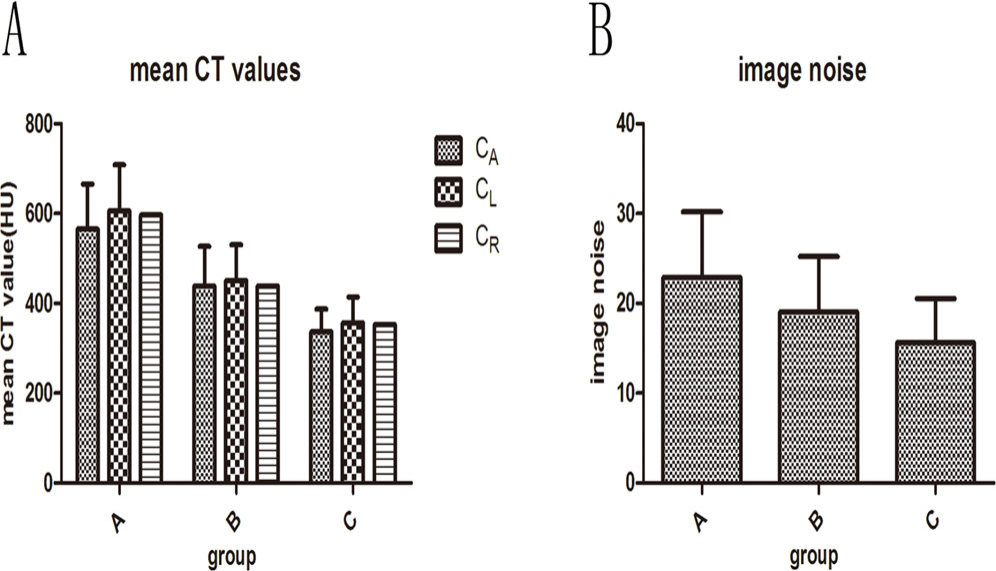
Objective image quality data. Objective image quality data of three groups, including mean CT attenuation values (A), image noise (B), CNR and SNR. Fig. 2A and 2B show that mean CT attenuation values and image noise decrease as kVp increases(P<0.05).

The results for three groups in terms of objective image quality were reported in Table 1 and Table 2. The mean CT attenuation values of AR (C_A_), LMA (C_L_), RMA (C_R_) and image noise (N) were recorded to calculate CNR of AR (CNR_A_), LMA (CNR_L_), and RMA (CNR_R_) as well as SNR of AR (SNR_A_), LMA (SNR_L_), and RMA (SNR_R_). Fig. 2 demonstrates that there were significant differences among the three groups in terms of mean CT attenuation values of AR (C_A_), LMA (C_L_), RMA (C_R_) and image noise (N) (P<0.05). We found that there were no significant differences among the three groups in terms of CNR of AR (CNR_A_), LMA (CNR_L_), and RMA (CNR_R_) (P > 0.05) as well as SNR of AR (SNR_A_), LMA (SNR_L_), and RMA (SNR_R_) (P > 0.05).

### Subjective image quality analysis

**Fig 3:**
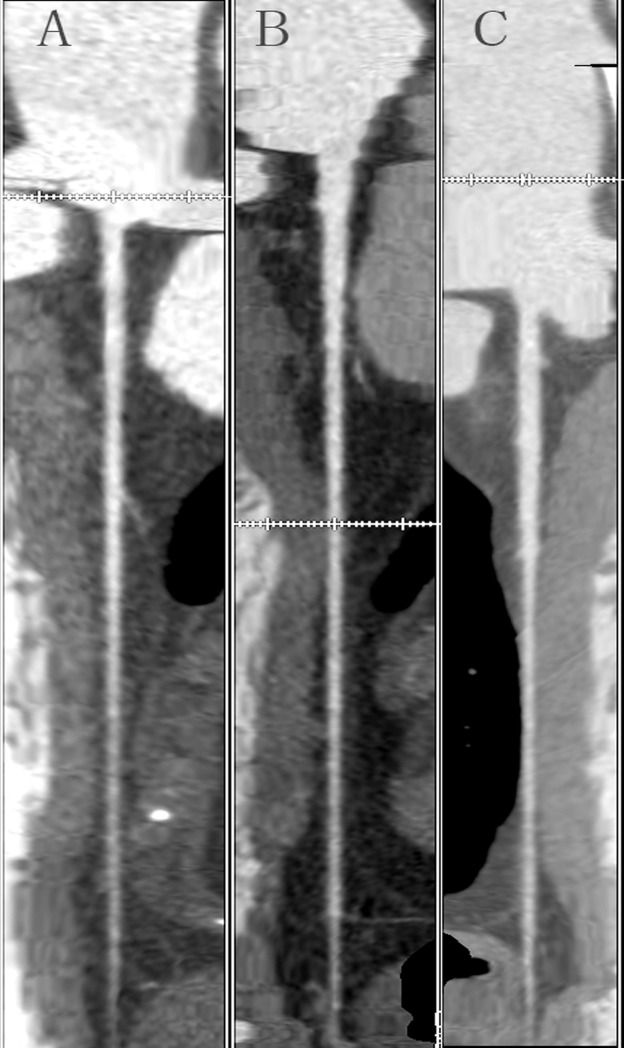
CTA of left anterior descending coronary artery in three patients using three protocols. A, 80 kVp, 300 mgI/ml; B, 100 kVp, 300 mgI/ml; C, 120 kVp, 370 mgI/ml with the same score: 4.30 rated by radiologists, which could be regarded as the same image quality.

**Fig 4:**
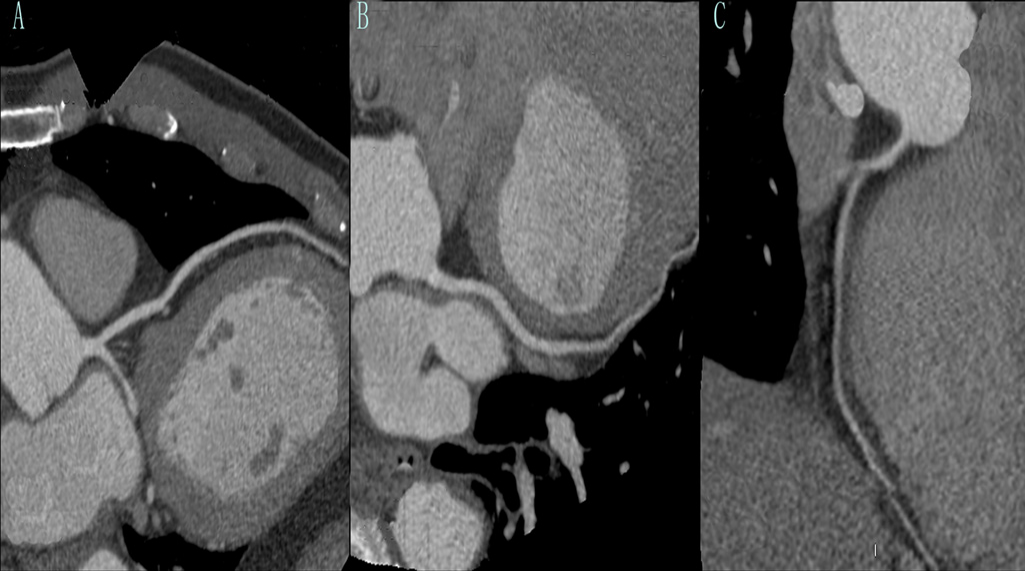
One example. A 54-year-old man with a history of chest pain underwent CT coronary angiography (curved MPR with 100kVp, protocol) and BMI of 26.4 kg/m^2^. We delivered the contrast medium (300 mgI/ml). A: left anterior descending; B: left circumflex; C: right main artery. The radiation dose was 2.45mSv. We can clearly make the diagnostic decision according to this figure: there is no calcified plaque or stenosis in left anterior descending, left circumflex and right main artery.

The results for the three groups in terms of subjective image quality are reported in Table 3. We found that there were no differences among the three groups in terms of subjective image scores (P > 0.05). Fig. 3 and Fig. 4 are the examples of the CT coronary angiography with different parameters. We chose three patients’ images (Fig. 3). Three patients’ left anterior descending use three protocols: A, 80 kVp, 300 mgI/ml; B, 100 kVp, 300 mgI/ml; C, 120 kVp, 370 mgI/ml with the same image quality score: 4.30. A 54-year-old man with a history of chest pain underwent CT coronary angiography (curved MPR with 100kVp, protocol) and BMI of 26.4 kg/m^2^. We delivered the contrast medium(300 mgI/ml). The radiation dose was 2.45mSv. We can clearly make the diagnostic decision according to Fig. 4: there is no calcified plaque or stenosis in left anterior descending, left circumflex and right main artery.

**Table 3:** Subjective image scores of three groups in terms of all coronary artery segments.

Coronary artery segments	A	B	C	P value
overall	4.10±0.41	3.90±0.48	4.04±0.36	0.18
LMA	4.15±0.59	4.00±0.70	3.93±0.47	0.29
LAD				
Proximal	4.39±0.57	4.19±0.58	4.26±0.54	0.39
Mild	4.17±0.59	4.06±0.57	4.11±0.59	0.72
Distal	3.77±0.56	3.74±0.62	3.83±0.59	0.71
LC				
Proximal	4.21±0.50	3.85±0.63	3.90±0.72	0.06
Distal	3.87±0.50	3.54±0.63	3.65±0.80	0.07
RMA				
Proximal	4.35±0.55	4.10±0.62	4.44±0.57	0.06
Mild	3.95±0.69	3.82±0.63	4.12±0.50	0.08
Distal	4.04±0.62	3.82±0.64	4.11±0.56	0.10

## Discussion

Many researchers are focusing their efforts to minimize risks from radiation and contrast medium while maintaining acceptable image quality during the non-invasive assessment of CHD. CT coronary angiography can be achieved by prospective or retrospective scanning, although the former confers a reduction in the radiation dose and is therefore preferred.

Our results prove the feasibility of using low concentration contrast medium with a low kVp for prospectively gated CT coronary angiography. There are variables that affect coronary artery vascular imaging, such as height, BMI, breathing and contrast agents concentration used. On the premise of guaranteeing image quality, reducing the concentration of the contrast agents and the radiation dose is of great significance. In our study, we demonstrate that using 80 kVp and 100 kVp contributed to a 47–69% reduction in the radiation dose and higher CT attenuation of arteries, which are consistent with the results reported by Faggioni et al [24]. A potential explanation for the higher CT attenuation brought by lower kVp is the photoelectric effect [25]. The effect of low doses of radiation on CT coronary angiography will likely be debated for years to come, but we must follow the principle of the lowest effective dose: using the least amount of radiation to meet the needs of diagnosis, while avoiding unnecessary repeated radiological examinations. In addition, we demonstrate that 300 mg iodine/mL can meet the requirements for diagnosis with respect to enhancement and artifacts on CT coronary angiography according to our study, similar to the report by Seung Chai Jung et al [26] and Annemaieke [27].

According to previous research reports, CT coronary angiography can distinguish different densities of plaque [28]. Additionally, the accuracy of plaque density measurement is affected by the size of the partial volume effect, contrast medium concentration and the size of the plaques [29]. Furthermore, gemstone CT is able to achieve better imaging of coronary angiography and to assess the calcification of the coronary artery stenosis accurately. When the concentration of contrast medium is higher, small plaques may be masked, beam-hardening artifacts will also be produced and the image quality may be degraded according to the image points. However, a lower concentration of contrast medium will blur the main coronary angiography, which affects the observation and measurement of the plaques. Because different degrees of these effects can be produced after injection of iodine contrast medium, the higher the concentration of medium, the more prevalent the side effects appear in the resultant images. Moreover, iodine contrast medium itself is harmful to the body, occasionally causing a fluctuation of the heart rate, atrial and ventricular fibrillation and even kidney damage. It is reported that contrast-induced nephropathy is second only to aminoglycoside antibiotics in acute renal failure induced by drugs [30]. Thus, the use of low concentration contrast medium is suggested to avoid or decrease the possibility of adverse effect caused by contrast medium.

For patients undergoing CT coronary angiography, the radiation dose is a major concern. Recent literature reports that lowering the setting from 120 to 80 kVp for pulmonary CT angiography in adult patients has been reported to provide an average dose reduction of 40% [31–33]. Therefore, we think it is possible to lower the voltage for CT coronary angiography to reduce the radiation dose. Conventional CT protocols (such as 120 kVp) lead to higher radiation doses for slim patients compared with normal or over-weight patients because absorption of X-rays is higher in slim patients. This is particularly true for CT coronary angiography [34]. The use of 80 kVp has been successfully used for coronary studies, especially in slim patients and children [35], which is consistent with our results. Our study is designed to enable the comparison between the objective and subjective image quality of two sets of images per patient. As expected, an increase in CT attenuation values resulted from lower voltage, but CNR and SNR were not significantly different. An additional merit of lowering voltage should also be considered, namely the possibility of reducing the iodine load [18]. This, however, is beyond the scope of this study. The most important thing is that the lower voltages did not affect subjective image scores and that patients suffered less radiation, which means radiologists can use low voltage kVp protocols in daily clinical routine: when patients’ BMI<23, we can use the protocol of 80 kVp with 300 mg iodine (I)/ml, or we can use the protocol of 100 kVp with 300 mg iodine (I)/ml to avoid unnecessary radiation and absorption of iodine.

However, our study is of limited scope. First, the small number of patients surveyed in each group may not be representative of the greater population. Although over 100 patients were enrolled in this study, each group contained only 31–36 subjects for the data analysis. Accurate and representative results must be achieved by the study of a larger patient population. Follow-up studies are also needed to confirm this result. Second, this study does not include coronary angiography, which is the gold standard for diagnosis of CHD and evaluation of diagnostic accuracy. Third, only two different iodine concentrations were used. With a limited difference in iodine concentration, the exact difference between the concentrations tends to be ignored. Whether a concentration that is lower than 300 mg I/ml with proper voltage can achieve the same image quality requires a further clinical trial. Finally, we did not take patients with BMI ≥ 28 into consideration in our study, meaning that lowering the concentration of contrast medium and voltage was not examined for BMI-related effects. Additionally, injection protocols (injection rate and volume injected) were set previously and their statistical influence on image quality was not determined. Further evaluation is required to determine whether image quality can be maintained using low concentration contrast medium and reduced voltage in patients whose BMI ≥ 28.

In conclusion, the image quality using low concentration contrast medium associated with low kVp is excellent and comparable to 120 kVp in CT coronary angiography. Thus, we can use a protocol of a low concentration of contrast medium and radiation dose to obtain the same quality of CT coronary angiography in order to reduce the adverse effects as much as possible.
